# Evolution in intergenerational exchanges between elderly people and their grandchildren in Taiwan; data from a multiple round cross-sectional study from 1993 to 2007

**DOI:** 10.1186/1471-2458-11-639

**Published:** 2011-08-11

**Authors:** Feng-jen Tsai, Sandrine Motamed, Nadia Elia, André C Rougemont

**Affiliations:** 1 Institute of Social and Preventive Medicine, University of Geneva 1, rue Michel Servet, CH-1211 GENEVE 4, Switzerland; 2 Fellow of the National Science Council, 106, Sec. 2, Heping E. Rd., Taipei 10622, Taiwan, R.O.C

## Abstract

**Background:**

This study aimed to evaluate social evolution in Taiwan in recent decades using the changing pattern of care provided by grandparents for their grandchildren as an indicator.

**Methods:**

Data from the second, fourth and sixth wave surveys of the *Study of Health and Living Status of the Middle-Aged and Elderly in Taiwan *were used for the analysis. This survey collected individual characteristics, including age, gender, education, ethnicity, dwelling place, living with partners, co-resident with children, employment status, self-reported health status and their provision of care for their grandchildren. Information about the attitudes toward National Health Insurance (NHI) was further collected in a questionnaire of 1999 following the implementation of NHI in 1995. By elders, we mean persons 60 or more years old. By grandchildren, we mean persons under 16 years of age. First, changes in individual characteristics were compared during these study periods (chi-square test). Then the logistic regression was performed to determine how significantly elders' grandchild-care behavior was associated with their individual characteristics.

**Results:**

The percentage of elders providing grandchild care increased from 7.7% in 1993 to 13.6% in 1999, and then to 19.4% in 2007. By analysis, significant association was found between behavior in taking care of grandchildren and individuals of lower age, grandmothers, those living with partners or co-residing with children, those unemployed and those with better self-reported health status. And the effect of year was confirmed in the multivariable analysis.

**Conclusions:**

This study pointed out the changing pattern of elders' behavior in taking care of their grandchildren as the main indicator and their related individual characteristics. We argue the need for improving social security policies in an ageing society. We suggest that the interaction between population policies and those of social welfare, including policies for health care and childcare, should be carefully evaluated.

## Background

Together with low fertility rates and a longer life expectancy, the rapid ageing of populations is a global phenomenon which requires a research and policy response concerning the potential consequences of this important demographic evolution [1,2]. The "generational contract" is the most important and contentious dimension of contemporary welfare systems for an ageing society. The key issue presented by population ageing is "to protect the old and invest in the young while keeping a balance between financial sustainability and the principles of social justice and fairness" [3]. However, most of the discussion focuses on its public (state and market provided) dimension, namely retirement pension systems and health and long-term care policies [4]. The importance of the private (family) dimension, which refers to the transfer of money and help between generations in the family, as well as the interaction between the public and private dimensions for an intergenerational contract have not been discussed sufficiently.

The social evolution and changing patterns in intergenerational transfers are multi-dimensional phenomena resulting from complicated factors. Instead of describing all the changes in intergenerational transfer, we focused on a single indicator, namely "child care provided by the grandparent" as the core component demonstrating the evolution in the intergenerational contract. Child care provided by their grandparents covers various contexts, both physical and mental. Some clear examples of physical care include taking grandchildren to school and picking them up again, or cooking and doing the laundry for them; mental support includes taking an active interest in their life and reading books for them. Typical and common arrangements for child care provided by grandparents are baby-sitting over the weekend or during the evening, looking after children when their parents are at work, or taking care of grandchildren under other circumstances on a regular or irregular basis [5]. Studies in the United States focusing on the intensity of grandchild care provided by grandparents during the child's first three years found that grandparents provided child care more sporadically when mothers were relatively young and worked nonstandard hours but provided extended full-time grandparent care for mothers with more extensive full-time employment [6]. These both reflect the parents' need for their own parents' assistance in taking care of grandchildren in the changing society.

The role of grandparent was pointed out to be central to the model of intergenerational solidarity, and taking care of their grandchildren has been identified as a particularly important form of multigenerational family support [7,8]. Theoretically, exemplary factors affecting intergenerational family transfer include structural factors such as demographic structure of families and labor-force structure, institutional factors like family and social security policies as well as cultural factors such as family and gender value [9]. It appears that younger, healthier elders with sufficient resources and time are more willing to take care of their grandchildren [3,10]. Further, previous studies not only suggested that grandparents' provision of grandchild care was driven by the availability and willingness of grandparents and the needs of parents and grandchildren [11], but also described the influence of grandparents' socio-demographic characteristic and availability, including partnership status and co-residence with their children, on elders' behavior in taking care of grandchildren [12-15]. In addition, studies comparing European countries have found the institutional social welfare system has a significant impact on grandparents' provision of grandchild care [3,16]. However, the changing pattern and the interaction between social welfare systems (public dimension) and intergenerational transfer concerning grandparents' provision of child care (private dimension) has not yet been evaluated in Asian countries.

Taiwan is a typical Chinese society which is strongly influenced by the traditional value of filial piety. Individuals are brought up to take the responsibility of extending the family generations and to have frequent intergenerational transfers. The culture not only emphasizes the responsibility of adult children to take care of their elders, but also addresses the old people's wish and willingness to have and to take care of grandchildren [17,18]. With a decreasing fertility rate (from 1.81 in 1990 to 1.68 in 2000 and then to 1.1 children per woman in 2006) and increasing life expectancy (from 71.6 years for men and 77.6 years for women in 1993 to 75.09 years for men and 81.90 years for women in 2007), ageing is an emerging issue - in the near future (2026) in Taiwan the old-age population will be over 20% [19]. Combined with the social evolution brought about by rapid industrialization and urbanization during the 20th century, the pattern of intergenerational transfer in Taiwan is changing. For example, fewer and fewer elders live with their children and more elders hold the idea that they should rely on themselves for their future life [19]. Moreover, the popular requirement and the recognition of the government's responsibility to offer welfare systems, including universal health coverage for the people and minority groups, also became apparent during this period [20]. However, the pattern of elders' behavior in taking care of grandchildren was poorly documented concerning these social changes. The possible effect of the social welfare system on it was also lacking. The aim of this study is to investigate the changing pattern of grandchild care provided by grandparents in Taiwan. Our hypothesis is that society has evolved over time in that elderly adults take more care of their grandchildren than before. To do so we used the data from the Study of Health and Living Status of the Middle-Aged and Elderly in Taiwan.

## Methods

### Participants and Survey Design

The data used in this study are from the second, fourth and sixth wave surveys of the Study of Health and Living Status of the Middle-Aged and Elderly in Taiwan, a longitudinal, multidisciplinary national survey representing the population of individuals aged 50 and over in Taiwan. The first survey of the study was conducted in 1989 on people aged 60 and over. The following surveys were conducted every 3 years. To maintain the representation of people aged 60 and above and to increase the elderly population to individuals aged over 50, the second cohort was included in the second survey in 1996 and the third cohort was included in the fifth survey in 2003. The data were openly available on request to the Bureau of Health Promotion, Department of Health, R.O.C (Taiwan). We used information from the second, fourth and sixth waves of the study which were conducted in 1993, 1999 and 2007. A total of 3,155 individuals aged 64 and over were in the sample of the second wave survey in 1993, the sample of the fourth wave survey in 1999 was of 4,440 persons aged over 53, and the sample in 2007 was of 4,534 persons aged 54 and over. For comparability, only individuals of 60 years old and older were included in our analysis. In order to examine elderly behavior in taking care of grandchildren, only those who had grandchildren were included in this study. In addition, if the individual repeatedly joined in more than one wave surveys, only the information of the last wave was used in the study. The final analytic samples in this study were therefore 868 elders in 1993, 1,876 in 1999 and 2,520 in 2007. As a guide to the size of the samples, in 1993 there were just over 2.2 million people aged over 60 (10.58% of Taiwan's 23 m population) while in 2007 over 3.1 million were in this age group (13.64% of the population) [21].

Individual characteristics related to elderly behavior in taking care of grandchildren used in the study include age, gender, education, ethnic group and living place. In addition, information about their partnership status (living with partners or not), employment status and self-reported health status were used in the analysis as well as whether they were co-resident with their children.

To evaluate behavior in taking care of grandchildren, elders were asked "whether they currently looked after their grandchildren for their children". In the questionnaires of 1999 and 2007, the frequency of taking care of grandchildren as "usual" and "sometimes" was also collected. In order to compare this with the data in 1993, answers of "usual" and "sometimes" were classified as "yes" in this analysis.

The self-reported health status of elders was collected with the question "how do you feel about your current health status?" The multiple response answer was scaled on 5 levels ranging from "very good" to "very bad". Following Taiwan's implementation of National Health Insurance (NHI) in 1995, elders' attitudes toward NHI were further collected four years later in the 1999 survey. These attitudes toward NHI were collected as answers to the question: "After implementing the NHI, are you satisfied with the current system such as insurance fee, co-payment and so on?" The multiple response answer was scaled on five levels, ranging from "very satisfied" to "very unsatisfied", with the additional possibility of "have no answer". A further question, "Do you feel the NHI is helpful for securing your future health and medical care?", was asked. Its multiple response answer was scaled on three levels as "very helpful", "some help", "not helpful"; other possible answers were "have no answer" and "never think about this issue".

### Statistical analysis

Two approaches were used to analyze the data. For summary statistics, the chi-square test was used to compare between the 1993, 1999 and 2007 samples: age, gender, education, ethnic group, living place, partnership status, co-residence with their children, employment status, self-reported health status together with whether they took care of their grandchildren. A logistic regression model was then performed to determine, the association of elders' behavior in taking care of grandchildren and their characteristics for total participants. All observations with missing data on any variable considered for analysis were excluded from the multivariable model.

The odds ratios (ORs) and the 95% confidence intervals (CIs) and the significance level at 0.05 were provided by the software SPSS version 18.0.

## Results

### Individual characteristics

Individual characteristics of elders are shown in Table 1. The individual characteristics of "gender" and "ethnicity" were the only two non-significant factors but the other factors including age, education, living place, partnership status, co-residence with children, employment status and self-reported health status differed significantly between these three groups. There were 47% illiterate elders in 1993 but only 27% in 2007. However, the percentage of city-dwelling elders increased from 47% in 1993 to 51% in 2007. The prevalence of elders living with partners decreased from 52% in 1993 to around 30% in 2007, and the prevalence of living with their children also decreased from 68% in 1993 to 60% in 2007. Furthermore, the percentage of elders with no job increased from 85% in 1993 to 89% in 1999, but then decreased to 83% in 2007. However, the percentage of those with a part time job decreased from 5.88% in 1993 to 2.29% in 1999, then slightly increasing to 2.9% in 2007. In 1993, 73% elders reported their self-perceived health as good to normal. But only 58% of the sample reported their self-perceived health as good to normal in 1999, though the percentage increased to 67% in 2007. For elders' attitudes toward NHI evaluated in 1999, 77% of the elders felt satisfied with NHI and 74% of the elderly felt that their future health life would be secured by NHI.

**Table 1:** Personal Characteristics of elders

	Total		1993		1999		2007			
	n = 5264		n = 868		n = 1876		n = 2520			
			
Characteristics	N	%	N	%	N	%	N	%	chi-sq	p-value
Gender										
Male	2770	52.63	493	56.80	1009	53.78	1268	50.32	3.07	0.22
Female	2494	47.37	375	43.20	867	46.22	1252	49.68		
Age (years)										
60~69	1722	32.70	244	28.11	346	18.44	1132	44.92	1152.6	< 0.0001
70~79	2298	43.66	421	48.50	1075	57.30	802	31.83		
80~89	1106	21.01	188	21.66	402	21.43	516	20.48		
90~99	138	2.62	15	1.73	53	2.83	70	2.78		
Education										
Illiterate	1800	34.20	409	47.12	710	37.85	681	27.02	243.08	< 0.0001
Under high school	2847	54.09	346	39.86	949	50.59	1552	61.59		
College and above	273	5.19	29	3.34	89	4.74	155	6.15		
Can read	340	6.44	81	9.33	127	6.77	132	5.24		
Ethnicity										
Fuchien	3544	67.32	567	65.32	1232	65.67	1745	69.25	9.14	0.17
Hakka	894	16.99	162	18.66	307	16.36	425	16.87		
Mainlander	726	13.79	115	13.25	298	15.88	313	12.42		
Others	85	1.62	19	2.19	32	1.71	34	1.35		
Living place										
Large city	753	14.31	98	11.29	262	13.97	393	15.60	36.5	< 0.0001
Big city	661	12.56	127	14.63	236	12.58	298	11.83		
Small city	1196	22.72	181	20.85	412	21.96	603	23.30		
Township	1032	19.59	174	20.05	350	18.66	508	20.16		
Rural area	1622	30.82	288	33.18	616	32.84	718	28.49		
Partnership status										
Living without partner	1972	37.45	413	47.58	1113	59.33	1766	70.07	67.34	< 0.0001
Living with partner	3291	62.53	455	52.42	763	40.67	753	29.88		
Co-residence with children										
No	1914	36.35	274	31.57	642	34.22	998	39.60	53.7	< 0.0001
Yes	3350	63.65	592	68.20	1234	65.78	1522	60.4		
Employment status										
No job	4515	85.77	745	85.83	1675	89.29	2095	83.13	61.95	< 0.0001
Full time job	582	11.06	72	8.29	158	8.42	352	14.0		
Part time job	167	3.17	51	5.88	43	2.29	73	2.9		
Self-reported health status										
Bad to very bad	1851	35.17	235	27.07	791	42.16	825	32.7	110.14	< 0.0001
Good to normal	3291	62.53	633	72.93	1085	57.84	1695	67.3		
Attitude toward NHI										
Satisfaction										
Very satisfied to normal					1454	77.51				
Unsatisfied to very unsatisfied					154	8.21				
Secured for your future health life by NHI										
Not helpful					150	8.00				
Very helpful to some help					1398	74.52				
Others					117	6.24				

#including "have no answer" and "never think about this issue"

## missing data is not shown in this table

### Taking care of grandchildren

**Figure 1:**
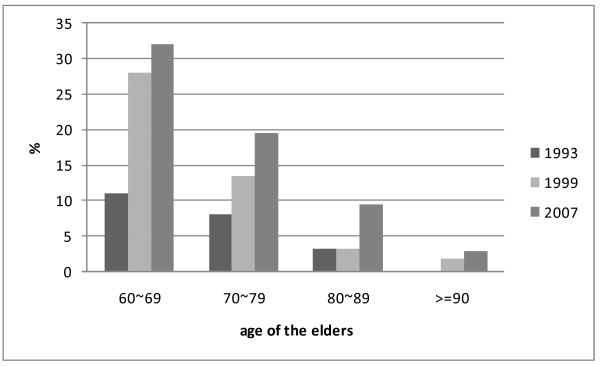
**Elders who take care of grandchildren (in percentages)**.

Reclassifying the answers "usual" and "sometimes" to "yes" in 1999 and 2007, the percentages of elders who took care of their grandchildren increased dramatically from 7.7% in 1993 to 13.6% in 1999, then to increased to 19.4% in 2007. Details stratified by ages are shown in Figure 1. For elders in their sixties, the percentage of grandchild-carers increased rapidly from 11.07% in 1993 to 28.03% in 1999, and then to 32.07% in 2007. The prevalence of grandchild care by elders in their 70s slightly increased from 8.08% in 1993 to 13.40% in 1999, and then continuously increased to 19.45% in 2007. And for those in their eighties the provision of grandchild care increased from 3.19% in 1993 to 3.23% in 1999, then jumped up to 9.5% in 2007.

### Associations between elders' individual characteristics and the provision of grandchild care

The associations between elders' individual characteristics and their behavior in taking care of grandchildren are shown in Table 2. In general, grandmothers, those in the lower age bracket, living with partners, co-residing with their children, being unemployed and self-reporting better health status were all found to increase grandchild-caring behavior. In addition, the effect of year on elders' behavior as taking care of their grandchildren was confirmed in the multivariable analysis. Grandparent take more care of their grandchildren as time goes by even after adjusting for other factors.

**Table 2:** The odds ratios of personal characteristics and the provision of grandchild care by logistic regression

	Total
	
Characteristics	Odds ratios (95% CI)
Gender	
Male	--
Female	1.35(1.13-1.61)**
Age (years)	
60~69	--
70~79	0.43(0.36-0.52)***
80~89	0.14(0.10-0.20)***
90~99	0.08(0.02-0.24)***
Partnership status	
Living without partner	--
Living with partner	1.73(1.41-2.11)***
Ethnicity	
Fuchien	--
Hakka	1.19(0.96-1.47)
Mainlander	1.74(1.34-2.26)***
Other	0.95(0.47-1.93)
Living place	
Large city	--
Big city	1.06(0.77-1.45)
Small city	1.17(0.90-1.54)
Township	1.42(1.07-1.89)*
Rural area	1.16(0.89-1.51)
Co-residence with children	
No	--
Yes	2.64(2.19-3.19)***
Employment status	
No job	--
Full time job	0.51(0.39-0.67)***
Part time job	0.83(0.54-1.28)
Self-reported health status	
Bad to very bad	--
Good to normal	1.67(1.39-2.00)***
Year	
1993	--
1999	2.01(1.49-2.72)***
2007	2.34(1.75-3.14)***

*p value < 0.05 ** p < 0.01 ***P < 0.001

#including "have no answer" and "never think about this issue"

## Discussion

Our study results show the changing pattern of elders' behavior in taking care of grandchildren from 1993 to 2007. Over the given period the provision of grandchild care increased, especially for elders in their 60s and 70s. In detail, in 1999 elders in their sixties provided much more child care than those in 1993, and elders in their seventies provided much more child care in 2007 than before. Elders' age, gender, partnership status, co-residence with their children, employment status and self-reported health status were significantly related to their provision of grandchild care.

With industrialization and urbanization, the female labor force participation rate in Taiwan increased from 36.4% in 1960 to 49.6% in 2010 [22]. In the mean time, illiterate elders in Taiwan gradually became less frequent and more old people lived in urban than in rural areas. Moreover, more elders lived without partners and fewer and fewer lived with their children. Traditionally, the ideal realization of filial piety for the elders was to live in a three-generational household or live with adult children. However, the emerging nuclearization of both young and old generations is producing complicated intergenerational relations. With urbanization, more people worked and lived in cities. They might remain in the city after their retirement for its familiar living environment, better accessibility to medical care, and to live closer to their children so as to have closer contact between generations, including providing care for the children of their children. It might also be the reflection of elders' awareness of their future needs for care and help. Although the government will take the responsibility of the care of old people when they are completely incapable for taking care of themselves and lack sufficient resources, family members are the first priority for being care-givers at home. In order to receive help from their children in return, elders provided grandchild care even though it was more difficult to live with their children.

Furthermore, older adults without a job provided more grandchild care than elders with a full time job in our study result. However, more older adults had part time job in 1999 than elders in 1993 and 2007. This might result from the better health of the older adults with a part time job than those with no job in 1999 following Taiwan's implementation of National Health Insurance (NHI) in 1995.

The health care system is one of the important social welfare policies, especially for elderly people. Health care in Taiwan is administrated by the Department of Health, which introduced National Health Insurance on 1st March, 1995. Before NHI, there were several health insurance systems such as health insurance for government officers, farmers and laborers, but only 57% of the population was covered in 1994 [23]. With strong political backing, NHI was introduced in 1995 and the coverage rate then rapidly reached 97.62% for females and 94.11% for males in 1999 and then 99% by the end of 2004 [24]. As a single-payer compulsory social insurance plan, mainly financed through premiums based on a payroll tax, the insurance fee of NHI was controlled by the government and remained relatively low priced. As a compulsory insurance with a low insurance premium, NHI provided a possibility of an affordable non-employer based health insurance and a safe health environment for elders' future. This was supported by the old people's great satisfaction and feeling of security toward NHI (Table 1). With previous findings that older adults were more likely to work less or retire after receiving access to non-employer based insurance [24,25], the implementation of NHI and its comprehensive coverage rate might give the elderly more leeway to make their retirement decision and work-family balance. Therefore, elders might tend to retire to improve their family life, including taking care of their grandchildren. The significantly decreased elderly employment rate from 1993 to 1999 might also be supporting evidence for this explanation. Also, the result that mainlanders provided significantly more grandchild care might also support the argument. The term 'mainlanders' refers those who migrated with the Kuomintang (KMT) to the island of Taiwan from mainland China between 1945 and 1950, following the Chinese civil war. Because these elders had comparatively less family support in Taiwan, they had to work more for their future life security. Therefore, they might have felt less necessity to work after the implementation of NHI and so have a greater chance to devote more time to their family.

There was an interesting finding that the self-reported health of elders became worse after the implementation of NHI in 1999 and then became better in 2007. A possible explanation is that in order to save money elders might not have had a health examination before the implementation of NHI, but elderly people knew their real health status after the NHI's provision of a health check-up. Therefore, elders' self-reported health status in 1999 was worse than 1993. But then with the increasing understanding of healthier life styles and the improvement in medical techniques, the elder's self-reported health status became better in 2007.

There were some limitations in this study. First, our finding can only be considered an association rather than a cause because the information on elders' individual characteristics and their behavior in taking care of grandchildren was cross-sectional. Second, the age of the grandchildren was not specifically considered due to the lack of information in this study. Third, we believe we underestimated elders' provision of grandchild care in Taiwan for the high "reporting threshold"; those that stay with grandchildren for only part of a day might not be considered in Chinese culture as taking care of grandchildren. Fourth, the causal relationship between work and elder's behavior of taking care of their grandchildren is not clear.

## Conclusions

In conclusion, our study result illustrates the increasing prevalence of grandchild care provided by grandparents over time in Taiwan. Our finding does, nonetheless, suggest a complex interaction between governmental welfare systems and intergenerational family support in shaping the work-family life balance for older adults. We suggest that the interaction between population policies and those of social welfare, including health care and childcare policies, should be carefully evaluated. We would also encourage policy makers to widen their thinking concerning any particular social welfare policy, linking its specific purpose with other, surrounding, functions. Further studies are needed to evaluate the change of social welfare policies and its effect on intergenerational transfer in an ageing society.

## Competing interests

The authors declare that they have no competing interests.

## Authors' contributions

FJT carried out the design of the study, collected the data, performed the statistical analysis, interpreted the data, and drafted the manuscript. SM participated in formulating the study, interpreting the data, and helped to draft the manuscript. NE participated in the design of the analysis, interpretation of results, and in the revision of the final manuscript. AR participated in formulating the study, interpreting the data, and helped to revise the manuscript. All authors read and approved the final manuscript.

## Authors' information

Feng-jen Tsai holds a PhD in public health and an LLM degree in law. She is a lawyer in Taiwan and a postdoctoral fellow in the Institute of Social and Preventive Medicine University of Geneva.

Sandrine Motamed is MD, MPH. She is lecturer at the University of Geneva (Medical Faculty), chief resident at the University Hospital of Geneva and adjunct associate professor of University of Hokkaido, Japan.

Nadia Elia is MD, MSc Epidemiology from the Institute of Social and Preventive Medicine, University of Geneva.

Andre C Rougemont, MD, MPH is full professor and, since 1993, the Director of the Institute of Social and Preventive Medicine, University of Geneva.

## Pre-publication history

The pre-publication history for this paper can be accessed here:

http://www.biomedcentral.com/1471-2458/11/639/prepub
